# Pathogenesis of bat rabies in a natural reservoir: Comparative susceptibility of the straw-colored fruit bat (*Eidolon helvum*) to three strains of Lagos bat virus

**DOI:** 10.1371/journal.pntd.0006311

**Published:** 2018-03-05

**Authors:** Richard Suu-Ire, Lineke Begeman, Ashley C. Banyard, Andrew C. Breed, Christian Drosten, Elisa Eggerbauer, Conrad M. Freuling, Louise Gibson, Hooman Goharriz, Daniel L. Horton, Daisy Jennings, Ivan V. Kuzmin, Denise Marston, Yaa Ntiamoa-Baidu, Silke Riesle Sbarbaro, David Selden, Emma L. Wise, Thijs Kuiken, Anthony R. Fooks, Thomas Müller, James L. N. Wood, Andrew A. Cunningham

**Affiliations:** 1 Institute of Zoology, Zoological Society of London, London, United Kingdom; 2 Department of Animal Biology and Conservation Science, University of Ghana, Accra, Ghana; 3 Veterinary Services Department, Ministry of Food and Agriculture, Accra, Ghana; 4 Wildlife Division of the Forestry Commission, Accra, Ghana; 5 Department of Viroscience, Erasmus University Medical Centre, Rotterdam, The Netherlands; 6 Wildlife Zoonoses and Vector Borne Disease Research Group, Animal and Plant Health Agency, Addlestone, United Kingdom; 7 Institute of Virology, Medical University of Berlin, Berlin, Germany; 8 Institute of Molecular Virology and Cell Biology, Friedrich-Loeffler-Institut, Federal Research Institute for Animal Health, Greifswald, Island of Riems, Germany; 9 School of Veterinary Medicine, University of Surrey, Guildford, United Kingdom; 10 Department of Pathology, University of Texas Medical Branch, Galveston, Texas, United States of America; 11 Department of Veterinary Medicine, University of Cambridge, Cambridge, United Kingdom; Universidad Nacional Mayor de San Marcos, PERU

## Abstract

Rabies is a fatal neurologic disease caused by lyssavirus infection. People are infected through contact with infected animals. The relative increase of human rabies acquired from bats calls for a better understanding of lyssavirus infections in their natural hosts. So far, there is no experimental model that mimics natural lyssavirus infection in the reservoir bat species. Lagos bat virus is a lyssavirus that is endemic in straw-colored fruit bats (*Eidolon helvum*) in Africa. Here we compared the susceptibility of these bats to three strains of Lagos bat virus (from Senegal, Nigeria, and Ghana) by intracranial inoculation. To allow comparison between strains, we ensured the same titer of virus was inoculated in the same location of the brain of each bat. All bats (n = 3 per strain) were infected, and developed neurological signs, and fatal meningoencephalitis with lyssavirus antigen expression in neurons. There were three main differences among the groups. First, time to death was substantially shorter in the Senegal and Ghana groups (4 to 6 days) than in the Nigeria group (8 days). Second, each virus strain produced a distinct clinical syndrome. Third, the spread of virus to peripheral tissues, tested by hemi-nested reverse transcriptase PCR, was frequent (3 of 3 bats) and widespread (8 to 10 tissues positive of 11 tissues examined) in the Ghana group, was frequent and less widespread in the Senegal group (3/3 bats, 3 to 6 tissues positive), and was rare and restricted in the Nigeria group (1/3 bats, 2 tissues positive). Centrifugal spread of virus from brain to tissue of excretion in the oral cavity is required to enable lyssavirus transmission. Therefore, the Senegal and Ghana strains seem most suitable for further pathogenesis, and for transmission, studies in the straw-colored fruit bat.

## Author summary

Rabies is a neurologic disease that causes severe suffering and is almost always fatal. The disease is caused by infection with a virus of the genus Lyssavirus, of which 16 species are known. These viruses replicate in neurons, are excreted in the mouth, and are transmitted by bites. Dogs are the most important source of rabies for humans, but recently there is a relative increase in people contracting the disease from bats. To better understand the development of human rabies caused by these bat-acquired viruses, we need to study this disease in its bat host under controlled circumstances. To do so, we chose a naturally occurring lyssavirus–host combination: Lagos bat virus in straw-colored fruit bats. We compared three available strains of Lagos bat virus (all isolated from brains of this bat species) for their ability to mimic a natural infection. We used intracranial inoculation to ensure infection of the brain. All three strains infected brain neurons, resulting in fatal neurologic disease, however only two of the strains showed the ability to reach the site of excretion—the mouth—and were considered a suitable virus to use for further studies of this disease in bats.

## Introduction

Rabies is an almost invariably fatal disease caused by rabies virus (RABV) or other members of the *Lyssavirus* genus, in the family of *Rhabdoviridae* of the order Mononegavirales. Rabies is predominantly transmitted by carnivores, in particular dogs, and causes more than 59,000 human fatalities annually [1]. As terrestrial rabies in domestic and wild carnivores is being brought under control by vaccination in high and middle income countries, the role of bats as a source of human infection has become more evident [2–4]. Additionally, although rare, the transmission of rabies from bats to terrestrial carnivores has been demonstrated as a driver for the generation of outbreaks in terrestrial mammals, as has been reported several times in North America [5–7]. Singular spill-over events of lyssaviruses other than RABV from bats to terrestrial mammals also have been reported [8–13]. Despite their increasing importance, we know relatively little about the dynamics of lyssavirus infections in bats [14] and how, for example, pathogenesis might differ from that in carnivores. This makes it difficult to assess the zoonotic risk of bat lyssavirus infections.

Some aspects of the pathogenesis of lyssavirus infections in bats are known. This knowledge is mainly based on experimental infections with four lyssaviruses in natural bat reservoir hosts: rabies virus, Australian bat lyssavirus, and European bat lyssaviruses 1 and 2 [15]. These experimental infections have shown that—like rabies virus in carnivores—these lyssaviruses target the brains of bats [16–19] with infection typically leading to encephalitis and death [20–23].

Other aspects of the pathogenesis of lyssaviruses in bats are poorly understood, such as mechanisms of virus excretion and transmission, the nature and duration of clinical signs, and the duration of virus excretion. An important limiting factor in this pathogenesis research is that oral excretion of virus is an uncommon event in experimentally infected bats [20–41].

This study was an initial step to reach our overall goal of developing an experimental model that mimics natural lyssavirus infection in a natural reservoir bat host. With ‘natural reservoir host’ we mean a host that is naturally infected and has co-evolved with the pathogen. For the experimental model, we chose Lagos bat virus (LBV) and the straw-colored fruit bat (*Eidolon helvum*). Lagos bat virus, which is divided into four lineages, A to D [15], is endemic in the straw-colored fruit bat [42], a common and widespread bat species in sub-Saharan Africa, which is not considered as ‘Threatened’ by the International Union for Conservation of Nature (www.iucnredlist.org). Infections of LBV in mammals other than bats have been reported sporadically [11, 12, 43]. Infection of humans has never been demonstrated, but it should be noted that diagnostic analysis of human rabies cases in Africa, if undertaken, typically uses methods that do not distinguish RABV from LBV or other lyssaviruses [15]. While the impact of LBV on human health is currently unknown, the widespread distribution of the straw-colored fruit bat and the apparent high rate of exposure of this species to LBV across its range [44], the increasingly close association of people with this bat species [45] and the failure of rabies vaccination to immunize against LBV [46], indicate that this pathogen has the potential to be an important public health threat.

The specific goal of this study was to choose the most suitable LBV strain, out of three that were available to us, for further studies. All three strains originally had been isolated from the brains of straw-colored fruit bats [47–49]. These strains differ in passage history and ability to cause infection in laboratory animals [11, 50–54]. To determine their ability to cause in vivo infection in a reservoir species, we inoculated these LBV strains into the cerebrum of straw-colored fruit bats. The bats we used were obtained from our closed captive breeding colony [55]. For each of the three strains, we determined the rate of infection, associated clinical signs, cell tropism, and pathologic changes.

## Materials and methods

### Ethics statement

Experimental procedures were approved beforehand by the Wildlife Division of the Forestry Commission of Ghana, the Zoological Society of London Ethics Committee (license number WLE638) and the Institutional Review Board of Noguchi Memorial Institute for Medical Research, University of Ghana, Legon.

Bats were anesthetized with a mixture of ketamine (5 mg/kg body weight [bw]; ketamine hydrochloride 115.36 mg/ml, Fort Dodge Animal Health Ltd, U.K.) and medetomidine (0.05 mg/kg bw; Laboratories SYVA S. A., Spain). Bats were euthanized by exsanguination under anesthesia with ketamine (5 mg/kg bw) and medetomidine (0.05 mg/kg bw), followed by cervical dislocation.

### Virus preparation

Virus stocks of three LBV strains were prepared and titrated according to standard methods. [56] The first virus strain was a lineage A LBV isolated from a bat in Dakar, Senegal in 1985. [11, 49] This virus was kindly provided as a fourth passage in mouse neuroblastoma cells (N2A) by M. Lafon, Institut Pasteur, Paris, France, and originally given to Institut Pasteur by J.P. Digoutte as a seventh passage in mouse brains. The virus was propagated three times in baby hamster kidney (BHK) cells. It reached an infectious virus titer of 10^7.25^ median tissue culture infectious dose (TCID_50_) per ml. The second virus strain was a lineage B LBV isolated from a bat in Lagos Island, Nigeria in 1956 [48]. It had a very large, but unknown, number of passages, primarily in BHK cells (including the last four passages). It reached an infectious virus titer of 10^5^ TCID_50_ per ml. The third virus strain was a lineage A LBV isolated from a bat in Kumasi, Ghana in 2013 [47], and was propagated four times in BHK cells. It reached an infectious virus titer of 10^5.7^ TCID_50_ per ml. All viruses were diluted to obtain a final inoculum dose of 10^3.5^ TCID_50_ in 30 μl.

### Experimental set up

We inoculated each of the three available LBV strains into straw-colored fruit bats in order to find the most suitable virus strain for the development of a model for LBV infection in a natural reservoir host. Although not a natural route of infection, we chose intracranial inoculation because this is the most reliable method to infect the brain, by circumventing the need for the virus to spread from a peripheral inoculation site (e.g. skeletal muscle) to the brain. If we initially used peripheral inoculation and no CNS infection occurred, we would not be able to rule out that the virus was defective. Intracranial inoculation was performed by stereotactic surgery to minimize variation in the site of inoculation and to minimize damage to the brain. The course of infection in inoculated bats was followed until general paralysis was reached in order to provide maximum time for the virus to spread from brain to peripheral sites, including sites(s) of excretion, and to determine whether bats were able to survive infection.

### Bats

Bats were obtained from a captive breeding colony that is maintained in Ghana [55] that had been closed to new wild-caught bats since January 2010. The animals were held in a double-walled cage with a solid roof to prevent any direct or indirect contact with free-living bats. All bats born in this colony had tested negative for antibodies against LBV using a modified version of the fluorescent antibody virus neutralization test with a lineage B LBV as the challenge virus [56]. The bats used in this experiment were all captive-bred and again tested seronegative at the beginning of the study, which started in October 2013. A month prior to the inoculation, and throughout the study, each bat used in the experiment was housed individually in a wire-mesh cage (80 x 80 x 80 cm). These cages were suspended from the roof of the animal house and separated by approximately two meters of space and tin baffles to prevent direct physical contact between bats and indirect contact via droplets (urine, secretions, food, water). Bats received diced mixed fruits (e.g. mango, papaya, banana) and ad lib water, which were replaced every day. Twelve bats were randomly assigned to one of four groups (three in each). The age category (juvenile, adult) and the sex of each bat was assessed according to body size and development of the external reproductive organs (S1 Table). The bats in each of the three virus groups were inoculated intracerebrally with one of the LBV strains. The bats in the fourth (control) group were inoculated with 30 μl of cell culture supernatant harvested from uninfected BHK-21 cells.

### Stereotactic surgery

Bats were anesthetized and their heads were placed in a ‘U’ frame stereotactic instrument (David Kopf Instruments, 902 Dual model). For pain relief and prevention of secondary bacterial infections, each bat was given a subcutaneous injection of butorphanol (0.2–2 mg/kg bw), buprenorphine (0.05 mg/kg bw), metacam (0.2 mg/kg bw; Meloxicam 2 mg/ml, Boehringer Ingelheim Vet medica GmbH, Germany) and enrofloxacin (0.2 ml/kg bw; Baytril 2.5%, Bayer plc, U.K). The cranium was exposed by skin incision and application of skin retractors. A 0.8 mm diameter dental drill was used to make a perforation in the parietal bone, unilaterally, 2 mm to the right of the midline, level with the lateral canthi of the eyes (approximately A/P, -2 to -4 mm; M/L, 2 mm; D/V, 4 mm from the Bregma point). The inoculum (see above) was injected at a depth of 5 mm, in a volume of 30 μl using a Hamilton syringe with a 36-gauge needle at a speed of 5 μl/min. After injection, the needle was kept in place for an additional 4 min before slow withdrawal. The skin was stitched back in the original position. A transponder chip for individual identification and for measurement of body temperature was placed subcutaneously in the interscapular region (Bio Medic Data system Inc.). Anesthesia was reversed with atipamezole (0.1 mg/kg bw; Veterinary Essentials, U.K.). Bats were given oral enrofloxacin (0.6 ml/kg bw/day) and metacam (0.1 mg/kg bw/day) for three days after the day of the surgery.

### Clinical examination and sampling

Bats were initially observed twice daily, at 07.00 and at 16.00 GMT for the presence of clinical signs. After the first occurrence of clinical signs, frequency of observations was increased to every two hours, day and night. Observers were unaware of the group the bat was in. Oral swabs (individually wrapped 2.5 mm diameter cotton tip [Fisher Ltd.]) were taken twice per week until the onset of clinical signs, when they were taken daily until death. This resulted in oral swabs being taken one to three times per bat in the period of four days prior to death. Oral swabs were collected in RNAlater (Ambion) for viral RNA detection. Euthanasia was delayed to a time point of severe clinical signs (generalized paresis) in order to determine whether bats could survive LBV infection. The mock-inoculated bats were euthanized after the last virus-infected bat had died (day 9 post inoculation).

### Pathological examination

Necropsies and tissue sampling were performed according to a standard protocol as soon as possible (approximately 1 to 6 hours after death) within a class 2 biological safety cabinet in a biosafety level 3 laboratory. At necropsy, a standard range of tissues (see below) was collected (1) fixed in neutral-buffered 10% formalin for histological examination, (2) in plain dry tubes for virus isolation, and (3) in tubes with RNAlater for RNA extraction. The following 14 tissue samples were collected: brain (hippocampus, cerebellum, medulla oblongata in separate tubes), and arbitrarily selected pieces of salivary gland, tongue, heart, lung, liver, kidney, spleen, submandibular lymph node, duodenum, jejunum and colon. Each tissue was collected using a new pair of disposable forceps and a new scalpel blade on an individual gauze pad to prevent possible cross-contamination. For the tissues for histological examination, the formalin was replaced once after two to three days to enhance fixation; the samples were stored at room temperature. The samples in RNAlater were allowed to fix overnight at 4 °C, and then stored at -70 °C until analysis, whereas plain samples in dry tubes were flash-frozen at -70°C.

### Virological examination

A range of tests were performed in biosafety level 3 laboratories at the Animal and Plant Health Laboratory Agency, U.K. and at the Friedrich Loeffler Institute, Germany, as described below:

*Hemi-nested reverse transcription-PCR*. All tissues and oral swabs, were tested with hemi-nested reverse transcription PCR (hn-RT-PCR) according to a previously-described protocol [57]. This test has proven to be highly sensitive because amplification from a small number of target genomes is sufficient for detection [58, 59].

*Fluorescent antibody test* Touch impressions on glass microscope slides of the three different parts of frozen brain were made and stained with fluorescein-isothiocyanate (FITC)-conjugated anti-rabies mouse monoclonal antibody (Fujirebio Diagnostics, USA, anti-N) [56]. A rabies-virus-positive mouse brain was used as positive control. The brain of an uninfected mouse was used as negative control.

### Histology and immunohistochemistry

The formalin-fixed tissues were embedded in paraffin wax, cut in 4-μm-thick serial sections and routinely stained with hematoxylin and eosin to detect microscopic lesions. Immunohistochemistry was performed to detect lyssaviral antigen [60]. Briefly, tissue slides were prepared for immunohistochemical staining by leaving slides in hydrogen peroxide 30% for 20 min to block endogenous peroxidase. Antigen retrieval consisted of boiling in citric acid buffer for 10 min. Virus antigen was detected with a goat anti-rabies N protein IgG (Rabies polyclonal DFA Reagent Goat IgG FITC conjugate; EMD Millipore; 1:500) as primary antibody, and rabbit anti-goat IgG-HRP (DAKO; 1:200) as secondary antibody. The brain of a mouse infected experimentally with silver-haired bat rabies virus (kindly provided by P. Koraka) was included as a positive control. The test was performed with goat serum (1:500) not containing antibodies against RABV as a negative control. Immunohistochemistry was performed on all tissues collected. Six coronal brain sections were made at equal intervals from the rostral to the caudal ends of the fixed brains, to be able to compare antigen presence in similar levels of the brain areas between bats. These brain sections were scored for frequency of lyssaviral antigen expression without prior knowledge of the identity of the bats. For this, the number of antigen-positive neurons relative to the total number of neurons was estimated in each of 10 arbitrarily selected, 40X objective (Olympus BX51) fields per brain section. The average percentages per brain section were placed in the following categories: 0–25% (category 1), 26–50% (category 2), 51–75% (category 3) and 76–100% (category 4).

## Results

### Clinical signs

Bats in the Ghana and Senegal groups died or had to be euthanized earlier (four to six days after inoculation) than bats in the Nigeria group (8 days after inoculation) (Table 1). The time from onset of clinical signs to death was similar among all three groups: thus, although group sizes were small clear differences were observed between the three strains: the Ghana and Senegal strains had shorter incubation periods on average than the Nigeria strain. All virus-inoculated bats developed clinical signs only during the last 24 hours prior to death, with a rapid progression of neurological signs. Bats with clinical signs did not eat (9/9). The three groups differed in clinical presentation, although differences were not statistically significant due to the small group sizes. One of the initial clinical signs in the Senegal group was a marked sensitivity to external stimuli, such as approaching of the cage by a person, loud sounds, fast movement of an object (e.g. experimentator’s hand) near the cage (3 out of 3 bats), but this was not seen in any of the other groups. Muscle spasms were observed in the Senegal (3/3) and Nigeria (3/3) groups, but not in the Ghana group (0/3). Increased vocalization (3/3) and aggression (3/3) were seen only in the Ghana group. Foam around the mouth was seen in the Nigeria group (2/3) and in the Senegal group (1/3), but not in the Ghana group (0/3). The Nigeria bats showed lethargy throughout much of the clinical course of illness (3/3), but, if present, this was an agonal feature in the other bats. None of the bats experienced significant changes of body temperature except when they developed extreme paresis or paralysis at the terminal stage of the disease, when their body temperature decreased by 2–3°C compared to mock-inoculated bats. All mock inoculated bats remained clinically normal and survived until the end of the experiment (9 days post-inoculation). There was no apparent correlation between age or sex and any of the variables analyzed above.

**Table 1:** Presence (+) or absence (-) of clinical signs in bats inoculated with Lagos bat virus.

Inoculum Group	Bat no.	Days to death	Hindleg paresis	Wing paresis	Muscle tremors or spasms	Lethargy (not agonal)	Lip smacking	Foam around the mouth	Vocalization	Aggression	Hyperaesthesia^c^
Control	1	na^a^	**-**	**-**	**-**	**-**	**-**	**-**	**-**	**-**	**-**
	2	na	**-**	**-**	**-**	**-**	**-**	**-**	**-**	**-**	**-**
	3	na	**-**	**-**	**-**	**-**	**-**	**-**	**-**	**-**	**-**
Senegal	13	4	**-**	**-**	**+**	**-**	**-**	**-**	**-**	**-**	**+**
	5	6	**+**	**-**	**+**	**-**	**-**	**-**	**-**	**-**	**+**
	6^b^	5	**-**	**-**	**+**	**-**	**-**	**+**	**-**	**-**	**+**
Nigeria	7^b^	8	**+**	**+**	**+**	**+**	**-**	**-**	**-**	**-**	**-**
	8	8	**+**	**+**	**+**	**+**	**-**	**+**	**-**	**-**	**-**
	9	8	**+**	**+**	**+**	**+**	**-**	**+**	**-**	**-**	**-**
Ghana	10	5	**+**	**-**	**-**	**-**	**+**	**-**	**+**	**+**	**-**
	11	5	**+**	**-**	**-**	**-**	**-**	**-**	**+**	**+**	**-**
	12	6	**+**	**-**	**-**	**-**	**-**	**-**	**+**	**+**	**-**

^*a*^ na, not applicable

^b^ Spontaneous death (other bats were euthanized).

^*c*^ Increased sensitivity to external stimuli.

### Virological examination

All parts of the brain (hippocampus, cerebellum, medulla oblongata) of all virus-inoculated bats tested positive by both hn-RT-PCR and fluorescent antibody test for lyssavirus RNA and antigen respectively, while the brains of all mock-inoculated bats tested negative. The spread of lyssavirus to organs outside the brain (extra-encephalic), based on hn-RT-PCR testing of 11 extra-encephalic tissues per bat, differed substantially among groups (Table 2). In the Ghana group, extra-encephalic tissues in three bats tested positive, and these had 8 to 10 positive tissues per bat. In the Senegal group, three bats tested positive, but these had only 3 to 6 positive extra-encephalic tissues per bat. In the Nigeria group, only one bat tested positive, and it had 2 positive extra-encephalic tissues. Thus the Ghana strain disseminated to more extra-encephalic tissues than either of the other two strains.

**Table 2:** Distribution of Lagos bat viral RNA and antigen in tissues of experimentally infected bats.

Inoculum Group	Bat no.	Days to death	Tissue (lyssaviral RNA by hn-RT-PCR/lyssaviral antigen by immunohistochemistry)												Total no tissues positive hn-RT-PCR/ IHC
			Brain	Salivary gland	Tongue	Lymph node^a^	Heart	Lung	Duodenum	Jejunum	Colon	Liver	Kidney	Spleen	
Senegal	13	4	+ ^b^ /+	+/- ^c^	+/-	-/-	-/-	-/-	-/-	NA^d^	-/-	-/-	-/-	-/-	3/1
	5	6	+/+	+/-	-/-	-/-	+/-	+/-	-/-	-/-	+/-	-/-	+/-	-/-	6/1
	6	5	+/+	+/-	-/-^e^	-/-	+/+^f^	+/-	+/-	-/-	-/-	-/-	-/-	-/-	5/3
Nigeria	7	8	+/+	-/-	-/-	-/-	-/-^g^	-/-	-/-	-/-	-/-	-/-	-/-	-/-	1/1
	8	8	+/+	-/-	-/-	-/-	-/-	-/-	-/-	-/-	-/-	-/-	-/-	-/-	1/1
	9	8	+/+	-/-	-/-	-/-	-/-^g^	-/-	-/-	-/-	+/-	+/-	-/-	-/-	3/1
Ghana	10	5	+/+	-/-	+/-^h^	+/-	+/+^f^	+/-	+/+^i^	+/+^i^	-/-	+/-	+/-	+/-	10/5
	11	5	+/+	+/-	-/-	-/-	+/+^f^	+/-	+/+^i^	+/-	+/-	-/-	-/-	+/-	8/3
	12	6	+/+	-/+^j^	+/+^k^	-/-	+/+^f^	+/-	+/+^i^	+/-	+/-	+/-	+/-	+/-	10/5
Total positive			9; 9^l^	4; 1	3; 3	1; 0	5; 4	5; 0	4; 3	3; 1	4; 0	3; 0	3; 0	3; 0	

^*a*^ Submandibular lymph node.

^*b*^ +, Positive.

^*c*^ -, Negative.

^*d*^ NA, Sample not available.

^*e*^ Taste buds positive, neurons in ganglia not.

^*f*^ Neurons in ganglia of the heart contained lyssavirus antigen.

^g^ Ganglia were not visible in heart section.

^*h*^ Surface epithelium positive, neurons in ganglion not.

^*i*^ Neurons in myenteric plexi positive.

^*j*^ Neurons in ganglion in the interstitium of the salivary gland positive.

^*k*^ Taste buds and neurons in ganglia positive.

^*l*^ x; y, x indicates total number of RNA positives; y indicates total number of antigen positives.

All oral swabs tested negative by hn-RT-PCR. The virus was detected by hn-RT-PCR in salivary gland in all bats from the Senegal group, and in one bat from the Ghana group. However, virus was detected in just one salivary gland with immunohistochemistry, and this was in a bat’s salivary gland that had tested negative with hn-RT-PCR (see below).

### Pathological examination

**Fig 1:**
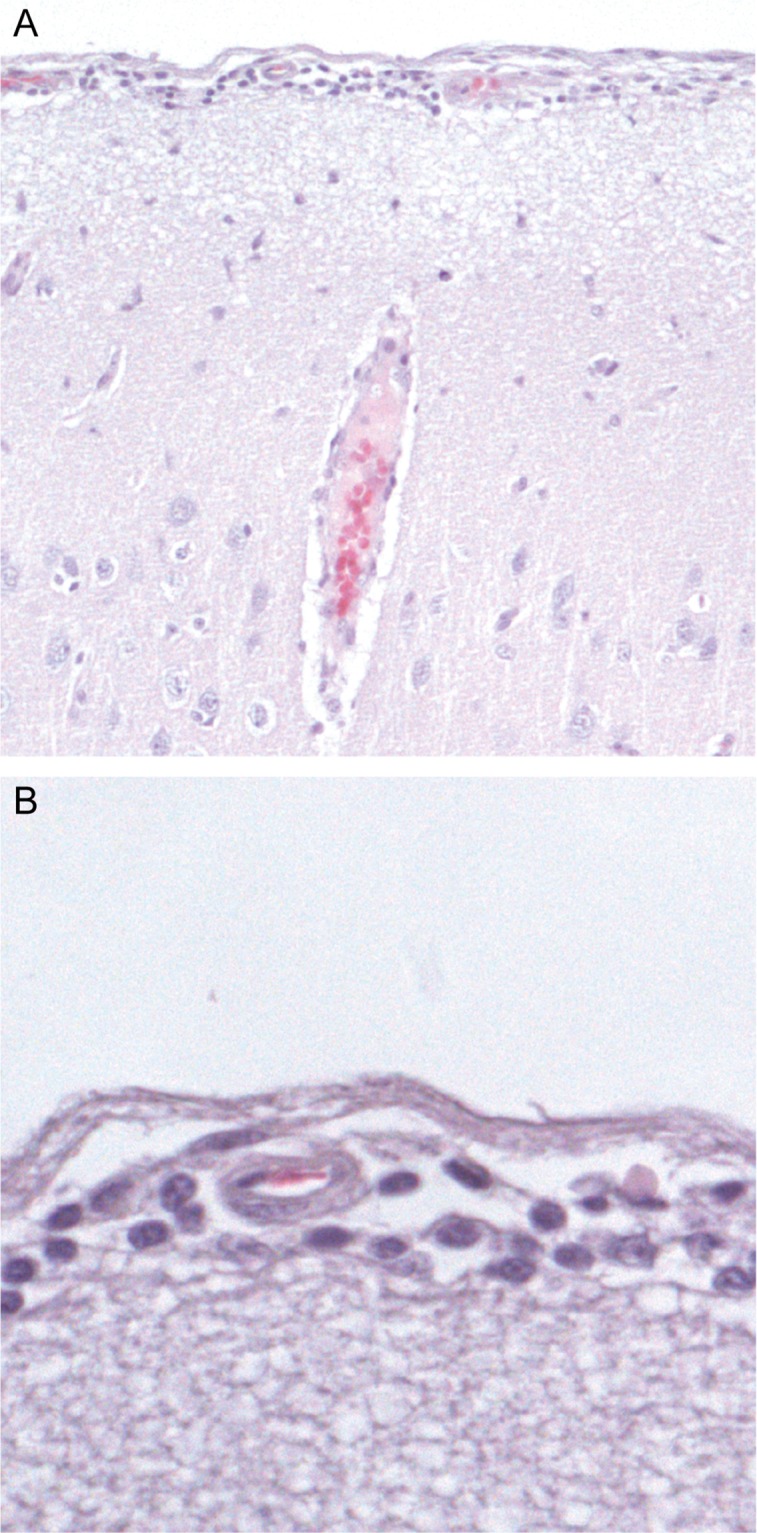
Inflammation in the meninges and brain parenchyma of a bat with Lagos bat virus infection (bat 10, Ghana strain). H & E staining. Original magnification: x10 (**A**), x40 (**B**) **A**: Perivascular cuffing surrounding blood vessels in the meninges. Cells surrounding blood vessels are mainly composed of small mononuclear cells with dense chromatin pattern (lymphocytes). **B**: Higher magnification of **A**.

At necropsy, no gross lesions were observed in any of the virus-inoculated or mock-inoculated bats. On histopathological examination, all virus-inoculated bats had diffuse, mild to moderate meningoencephalitis. This was characterized by the presence of a few to moderate number of lymphocytes in the meninges, around blood vessels (Fig 1). In the brain parenchyma, there was a mild increase in the number of glial cells compared to the mock-inoculated bats, and occasional pyknotic or karyorrhectic cells of undetermined origin (ranging from one to eight per five 40X objective fields). There were perivascular lymphocytic infiltrates, of up to three cell layers thick, in most brain sections. Negri bodies were not observed. Outside the brain, the only nervous tissue lesion seen was in bat 12 (Ghana group): the wall of the colon had a mild lymphocytic infiltration surrounding a partly necrotic myenteric plexus (plexus of Auerbach), of which the remaining neurons were not positive by immunohistochemistry (see below). The colon of this bat, however, did test positive for lyssavirus by hn-RT-PCR (Table 2).

There were a few lesions in bats 6, 8 and 11, which we considered to be incidental and not directly caused by LBV infection. Bat 6 (Senegal group) had an acute, mild, multifocal fibrinopurulent pneumonia, characteristic of aspiration pneumonia. This bat was noted to have had paresis and muscle spasms during the terminal 24 hours of infection, which could have caused aspiration of water. This lesion, therefore, might have been indirectly caused by the LBV infection. Both bat 6 and bat 8 (Nigeria group) had a chronic, mild, multifocal lymphoplasmacytic interstitial nephritis. Bat 8 had an acute mild focal necropurulent hepatitis. Bat 11 (Ghana group) had deeply eosinophilic, homogeneous to laminated, irregularly shaped structures (interpreted as sialoliths) in the lumina of about half of the secretory ducts of the salivary gland. Occasionally these structures compressed the lining ductular epithelial cells. None of the mock-inoculated bats had histological lesions in any of the tissues examined, including the brain.

### Immunohistochemistry

**Fig 2:**
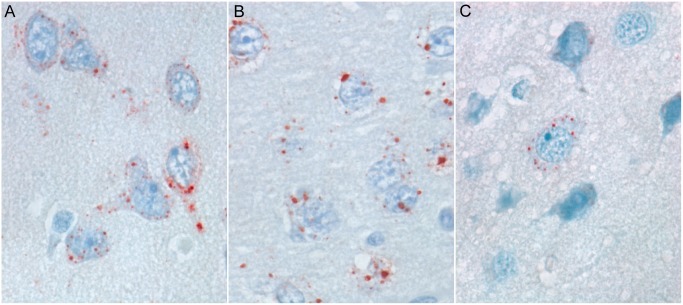
Lyssavirus antigen expression in neurons infected with different strains of Lagos bat virus. All sections immunostained for lyssavirus antigen. Original magnification: x100 (**A**, **B** and **C**). **A**: Senegal strain, relatively large number of neurons are infected, small cytoplasmic antigen (red) granules, infected neurons have variable numbers of granules (Bat 6). **B**: Nigeria strain, relatively large number of neurons are infected, large cytoplasmic antigen (red) granules, infected neurons have similar numbers of granules (Bat 9). **C**: Ghana strain, relatively few neurons infected, small cytoplasmic antigen (red) granules (Bat 12).

Cells positive for immunohistochemical staining, and thus positive for lyssavirus antigen, were found in a number of tissues. In most of these tissues, positively-stained cells could be clearly identified as neurons based on their morphology. When detected in neurons, antigen was located in the cytoplasm, with staining consisting of small (pinpoint to approximately 2 μm diameter) or large (approximately 5 μm diameter) granules. The number of granules per cell ranged from one to numerous. Neurons with multiple antigen granules were often found adjacent to neurons that did not contain any staining. Variation in the size and number of granules per neuron differed among groups. Stained neurons in the Senegal and Ghana group bats contained only small granules, with a high variation in the number of granules per neuron. In contrast, stained neurons in the Nigeria group bats contained large granules with little variation in the number of granules per neuron (Fig 2). Overall, the Ghana group had significantly fewer antigen-positive neurons than the Senegal or Nigeria group bats (*p* = 0.004, paired *t*-test; Table 3).

**Table 3:** Distribution of Lagos bat virus antigen in the brains of experimentally infected bats.

Inoculum Group	Bat no.	Days to death	Category of antigen-positive neurons per brain section						Average category of antigen-positive neurons per brain	Average category of antigen-positive neurons per brain, per group
			A^a^	B	C	D	E	F		
Senegal	13	4	1^b^	1	2	1	3	3	2	
	5	6	2	1	2	1	1	3	2	
	6	5	3	4	3	3	3	3	3	2
Nigeria	7	8	3	2	1	1	4	2	2	
	8	8	1	1	2	1	3	2	2	
	9	8	4	3	3	3	2	2	3	2
Ghana	10	5	1	1	1	1	1	3	1	
	11	5	1	1	1	1	2	3	2	
	12	6	1	1	1	1	1	2	1	1*
Average			2	2	2	1	2	3		

^*a*^ Brain section A corresponds to the most rostral part, section F to the most caudal part of the brain.

^*b*^ Category 1: 0–25% of the neurons antigen positive in 10, 40X objective fields arbitrarily selected in this brain section, 2: 26–50%, 3: 51–75%, 4:76–100%.

* Significantly lower than the average score of Nigeria and Senegal groups (*p* = 0.004, paired *t*-test).

In the brain, antigen-positive neurons were generally concentrated in the most caudal brain section examined, otherwise there were no clear patterns within or between groups comparing percentage of neurons infected across the six brain sections examined for each bat (Table 3). We could not exclude that a small number of positive cells in the brain were of other cell types, possibly glial cells. The majority of antigen positive cells in the brain did not show any signs of degeneration or necrosis; a few neurons showed evidence of cell shrinkage and loss of Nissl substance.

**Fig 3:**
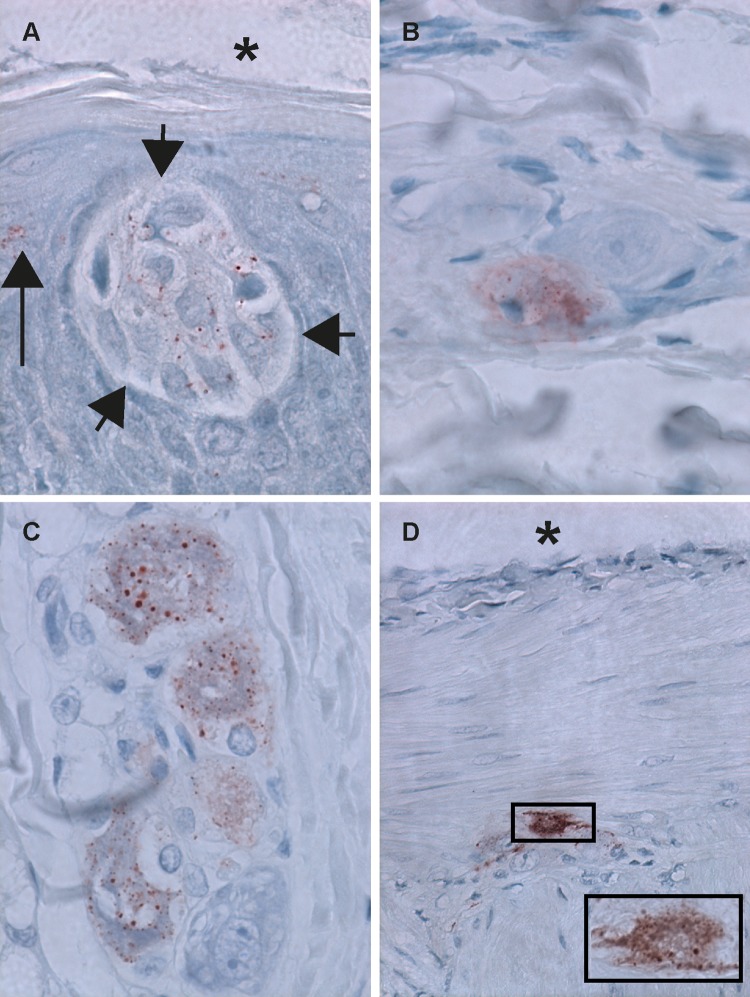
Lyssavirus antigen expression in neurons infected with different strains of Lagos bat virus. All sections immunostained for lyssavirus antigen. Original magnification: x 100 (**A**, **B** and **C**); x40 (**D**); x100 (inset of **D**) **A**: Taste bud (between short arrows) bordering the lumen of the oral cavity (indicated with asterisk) in cross section of tongue. Several neuroepithelial cells within the taste bud have small cytoplasmic antigen (red) granules. Epithelial cells directly adjacent to taste buds also occasionally have antigen granules (indicated with long arrow) (Bat 6). **B**: Three neurons in a ganglion within the connective tissue of a salivary gland. One neuron has small cytoplasmic antigen (red) granules (Bat 12). **C**: Neurons in a ganglion within the connective tissue of the epicardium (heart). One neuron has abundant small cytoplasmic antigen (red) granules. Directly adjacent one neuron with less antigen granules (Bat 6). **D**: Neurons within the myenteric plexus (intestine) have abundant small cytoplasmic antigen (red) granules. Asterisk indicates the serosa side of the intestinal wall (bat 11). Inset contains a higher magnification of the image in the square of **D**.

In the extra-encephalic tissues examined, lyssavirus antigen was detected in neurons. Antigen-positive neurons were found only in ganglia in salivary gland, tongue, and heart, and in myenteric plexi (also called Auerbach’s plexi) in duodenum and jejunum (Fig 3A–3C; Table 4). Submucosal plexi (also called Meissner’s plexi) were visible in all samples of duodenum, jejunum and colon, but no lyssavirus antigen was detected in their neurons.

**Table 4:** Neuronal routes different Lagos bat viruses travelled from the intracranial inoculation site to the periphery.

LBV strain(s)	Peripheral location	Deduced route for centrifugal spread of LBVs from intracranial inoculation site to periphery				
		Origin in CNS		Route (nerves & ganglia)	Characteristics of neuronal route	
		Specific nucleus	General location of nucleus in CNS		Number of synapses to pass	Division of nervous system involved
Ghana	Tongue ganglion	Salivatory	Motor medulla	Chorda tympani and glossopharyngeal nerves	1	Parasympathetic motor
Ghana	Otic ganglion (salivary gland)	Inferior salivatory	Motor medulla	Chorda tympani and glossopharyngeal nerves	1	Parasympathetic motor
Ghana, Senegal	Cardiac plexi at base of heart^a^	Dorsal motor nucleus of vagus nerve	Motor medulla	Vagus nerve	1	Parasympathetic motor
		Cranial four to five segments thoracic spinal cord	Spinal cord	Cervical paravertebral sympathetic trunk and postganglionic fibers	2	Sympathetic motor
Ghana	Myenteric plexi of duodenum and jejunum^a^	Dorsal motor nucleus of vagus nerve	Motor medulla	Vagus nerve	1	Parasympathetic motor
		Dorsal horn spinal cord	Spinal cord	Splanchnic nerve, celiac ganglion, and postganglionic fibers	2	Sympathetic motor
Ghana, Senegal	Taste buds on tongue	Solitary tract	Sensory medulla	Geniculate, petrosal and nodosal ganglia	2	Special sensory

^*a*^ More than one route possible because of innervation of the nervous plexi by both parasympathetic and sympathetic nervous system.

In tongue sections, lyssaviral antigen was detected not only in neurons but also in epithelial cells. Three bats had one to three foci of lyssavirus antigen-positive epithelial cells at the tongue surface (Table 2). In bat 6 (Senegal group) and bat 12 (Ghana group), the antigen-positive cells were part of a taste bud. This initiated more detailed investigation for the presence of taste buds in the tongue sections: the tongue section examined from bat 6 had three taste buds, two of which were antigen-positive, and that from bat 12 had one taste bud, which was positive. Three other virus-infected bats also had taste buds present in their tongue sections (bat 13, two taste buds; bat 7, five taste buds; bat 8, two taste buds), but none were lyssavirus antigen-positive.

Antigen was not detected in salivary gland epithelial cells of any virus-infected bat, although salivary gland sections were available for all nine. Antigen was not detected in any other tissues of the virus-infected bats, and was not detected in any of the tissues examined from the mock-inoculated bats.

## Discussion

Our results show that all three LBV strains are capable of infecting and replicating in the brain of the straw-colored fruit bat following intracranial inoculation. The predominant detection of virus in neurons confirms that LBV is neurotropic, similar to RABV and other lyssaviruses in other host species [61]. In many of our infected bats, virus antigen was detected in a large percentage (over 25%) of neurons at different levels throughout the brain at the time of death. The majority of infected neurons showed no visible pathological changes. The wide dissemination in the brain, an absence of severe neuronal lesions and the late development of neurologic signs suggest that, like RABV in other species [61], these LBV strains can replicate in neurons for some time before negatively affecting their function.

Virus spread from the site of intracranial inoculation to peripheral tissues was detected most frequently in bats infected with the Senegal and Ghana strains of LBV. The rapidity of spread from brain to the periphery—centrifugal spread—within a few days post inoculation, was likely due to the high viral dose inoculated directly into the brain. The extra-encephalic detection of lyssavirus antigen nearly exclusively in neurons indicates centrifugal spread via peripheral nerves, and fits with the accepted pathogenesis of lyssavirus infections [61], although the occurrence of viremia cannot be excluded [62, 63]. The higher frequency of PCR-positive results than immunohistochemistry-positive results in the peripheral tissues is likely due to the higher sensitivity of PCR. As a consequence, we only were able to detect immunohistochemistry-positive neurons when their cell bodies, aggregated in ganglia, were present in tissue sections. For this reason, immunohistochemistry and PCR results corresponded better in those tissues in which ganglia were usually present (heart, intestine). Centrifugal spread is necessary for lyssaviruses to reach the oral cavity, from where they can be transmitted to new susceptible individuals via bites. As we could not detect the Nigeria strain of LBV in the peripheral nervous system by immunohistochemistry, this strain is possibly not suitable for further pathogenesis studies. A difference between the Nigeria strain and the other two strains is the large number of in vitro passages it has undergone.

Although no marked lesions were present in the brain at the time of death, neuronal dysfunction was apparent from the neurological disease observed in our virus-inoculated bats. Although neurologic signs have not been previously described for LBV-infected bats, they correspond to those described previously for bats infected with other lyssaviruses [35, 64–66].

The apparent consistency of the clinical presentation seen within each infection group, and the differences among the bat groups, suggests a link with the virus strain used. Specifically, all three bats inoculated with the Senegal strain developed marked sensitivity to external stimuli, and all three bats inoculated with the Ghana strain developed aggression and excessive vocalization. Interestingly, the Ghana LBV was reported to have been isolated from an apparently healthy straw-colored fruit bat in the frame of a non-targeted virus isolation study in a large colony comprising ca. 350,000 animals in Kumasi, Ghana [47]. Here, aggression and vocalization may have been overlooked or misinterpreted. All three bats inoculated with the Nigeria strain developed general weakness, while this was only seen as an agonal feature in the other groups (Table 1). A correlation between specific clinical signs and virus strain used has been observed for other lyssavirus infections [67]. In general, the mechanism through which lyssavirus infection causes clinical signs is still a subject of debate. Lyssaviruses can have both direct and indirect effects on the functionality of neurons. These effects can be due to a change in neuronal electrophysiology, a change in the function of ion channels, a change in neurotransmission, a change in the amount of hormone production, or presumably other, as yet undefined, mechanisms. A combination of these effects might occur at specific anatomical locations explaining specific neuronal deficits in rabies patients. So far, no single or dominant mechanism has been identified [61].

All virus-infected, but no mock-inoculated, bats had a lymphocytic meningoencephalitis at death. This is similar to the histologic lesions seen in mice intracranially inoculated with lyssaviruses of different species [68]. The presence of lymphocytes in the absence of other cells, such as plasma cells or neutrophils, suggests that a Th1 or cell-mediated immune response occurred in these bats. This is surprising, as the direct inoculation of virus in the brain is not expected to incite an immune response at all: initiation of T cell responses are unlikely to occur within the central nervous system as naïve T cells do not cross the blood-brain barrier and lymph vessels and dendritic cells that are normally involved in picking up exogenous material are absent in the brain [69]. However, for rats and rabbits it has been shown that the grey matter interstitial fluid is drained via cervical lymph nodes, where antigen-presenting cells are triggered and where a subsequent adaptive immune response can develop [70]. It is also possible that some of the virus particles inoculated into the cerebrum in our bats entered the interstitial fluid, reached a lymph node, and triggered an adaptive immune response. It also is possible that the surgical procedure of intra-cranial inoculation damaged the blood-brain barrier, allowing virus to spill over into the blood, and immune cells from the blood to infiltrate the brain parenchyma, thus initiating a specific immune response. Whatever the mechanism of recruitment of lymphocytes to the brain in the virus-inoculated bats, due to our unnatural inoculation route this inflammatory response does not necessarily reflect the situation during natural LBV infection in the straw-colored fruit bat.

In our study, several neurons in ganglia and other cells outside the central nervous system were lyssavirus antigen-positive. We used this information to deduce via which nerves the different LBV strains travelled from site of intracranial inoculation to extra-encephalic tissues (Table 4). The Ghana strain travelled via parasympathetic motor neurons to reach the tongue and salivary gland ganglia. The Ghana and Senegal strains travelled via parasympathetic or sympathetic motor neurons, or both, to reach ganglia in heart and intestine. The Ghana and Senegal strains also travelled via special sensory neurons to reach taste bud epithelial cells. The Nigeria strain, however, even with a longer period of time for dissemination, did not travel via any of these routes.

For a lyssavirus to be suitable for use in pathogenesis studies, and specifically for causing infection in bats that leads to excretion of virus in the oral cavity, the virus needs to have the capacity to get to the excretion site. In straw-colored fruit bats that were naturally infected with LBV, salivary gland epithelial cells and tongue epithelial cells were shown to contain antigen [71]. These two cell types may thus be sites for virus excretion. Salivary gland epithelial cells are mostly innervated by parasympathetic motor neurons, and to a lesser extent by sympathetic motor neurons and by sensory neurons. Taste bud epithelial cells in the tongue are innervated by special sensory neurons. Although the salivary gland samples of some bats were positive by hn-RT-PCR, and the Ghana strain showed the capacity to infect neurons innervating the salivary gland, none of the infected bats in this study had antigen in salivary gland epithelial cells (Table 2). Possible reasons include the bats dying before the infection could reach the salivary gland epithelial cells, a different pattern of centrifugal spread to the salivary gland after intracranial inoculation than after natural infection, and the inability of our LBV strains to infect salivary gland epithelial cells. Some authors (e.g., [72, 73]) used viruses isolated from the oral cavity instead of from the brain for experimental infection studies; this may ensure that viruses are from a population that is able to infect cells important for excretion.

Although virus was found in surface epithelial cells of the tongue of two bats (bats 12 and 13), virus was not detected in any of the oral swabs. There are several possible explanations for this. First, immunohistochemistry to detect virus antigen in oral tissues may be more sensitive than hn-RT-PCR to detect viral RNA in oral swabs. Second, although there was virus in tongue surface epithelium, there may have been no excretion into the oral cavity. This does not fit well with what is known of the pathogenesis of other lyssaviral infections. Third, it may be that excretion was intermittent and swabs were taken at a time when virus was not excreted. Intermittent excretion has been proposed to explain alternating positive and negative results of serially collected oral swabs in other experimental lyssavirus infections in bats [20]. However, virus was not detected by hn-RT-PCR in any of our oral swabs. Fourth, there may have been loss of viral RNA in oral swab samples during transport or processing. This seems less likely as viral RNA could be detected in tissue samples that were similarly transported and processed. LBV has been detected in an oral swab of a naturally infected straw-colored fruit bat previously [71], showing that this is technically possible.

In conclusion, our study showed that intracerebral inoculation of each of three LBV strains into straw-colored fruit bats caused widespread infection of the brain, associated with meningoencephalitis, clinical signs consistent with rabies, progressing quickly to death of all virus-inoculated bats. Although group sizes were small, different clinical signs were observed and these were associated with the virus strains used. The distribution of virus antigen in extra-encephalic tissues indicated that the Ghana and Senegal LBV strains were able to utilize both sensory and motor neurons for dissemination to the periphery, and to infect epithelial cells of the tongue as a possible site of excretion. The Nigeria strain was rarely detected in any tissue outside the brain and may have lost the capability to use certain transport mechanisms in neurons, possibly due to multiple passages in cell culture. Based on the above results, both the Senegal and Ghana strains are suitable for further pathogenesis studies of LBV in the straw-colored fruit bat, a natural host of the virus. Now that we have shown that these strains are suitable, our next step will be to use a peripheral inoculation site, and thus a more natural route of transmission, for further pathogenesis studies in the straw-colored fruit bat.

## Supporting information

S1 Table Sex, age category and body weight of bats inoculated with Lagos bat virus or mock-inoculated (control). (XLSX)
